# 

**Published:** 1885-08

**Authors:** 


### CONTENTS.

### COMMUNICATIONS.

Oral Deformity........................................................ 377
Crown Work........................................................ 382
Address........................................................ 383

### PROCEEDINGS.

American Medical Association........................................ 386

### DENTAL LAW.

Delaware Dental Law ........................................ 403
Kansas Dental Law........................................ 405
Minnesota Dental Law........................................ 408
Wisconsin Dental Law........................................ 411
COMMENCEMENTS........................................ 415
SALMAGUNDI ........................................ 422

### EDITORIAL.

Obituary........................................ 426

